# High quality protein sequence alignment by combining structural profile prediction and profile alignment using SABERTOOTH

**DOI:** 10.1186/1471-2105-11-251

**Published:** 2010-05-14

**Authors:** Florian Teichert, Jonas Minning, Ugo Bastolla, Markus Porto

**Affiliations:** 1 Institut für Festkörperphysik, Technische Universität Darmstadt, Hochschulstr. 6-8, 64289 Darmstadt, Germany; 2 Centro de Biología Molecular "Severo Ochoa", (CSIC-UAM), Cantoblanco, 28049 Madrid, Spain; 3 Institut für Theoretische Physik, Universität zu Köln, Zülpicher Str. 77, 50937 Köln, Germany

## Abstract

**Background:**

Protein alignments are an essential tool for many bioinformatics analyses. While sequence alignments are accurate for proteins of high sequence similarity, they become unreliable as they approach the so-called 'twilight zone' where sequence similarity gets indistinguishable from random. For such distant pairs, structure alignment is of much better quality. Nevertheless, sequence alignment is the only choice in the majority of cases where structural data is not available. This situation demands development of methods that extend the applicability of accurate sequence alignment to distantly related proteins.

**Results:**

We develop a sequence alignment method that combines the prediction of a structural profile based on the protein's sequence with the alignment of that profile using our recently published alignment tool SABERTOOTH. In particular, we predict the contact vector of protein structures using an artificial neural network based on position-specific scoring matrices generated by PSI-BLAST and align these predicted contact vectors. The resulting sequence alignments are assessed using two different tests: First, we assess the alignment quality by measuring the derived structural similarity for cases in which structures are available. In a second test, we quantify the ability of the significance score of the alignments to recognize structural and evolutionary relationships. As a benchmark we use a representative set of the SCOP (structural classification of proteins) database, with similarities ranging from closely related proteins at SCOP family level, to very distantly related proteins at SCOP fold level. Comparing these results with some prominent sequence alignment tools, we find that SABERTOOTH produces sequence alignments of better quality than those of Clustal W, T-Coffee, MUSCLE, and PSI-BLAST. HHpred, one of the most sophisticated and computationally expensive tools available, outperforms our alignment algorithm at family and superfamily levels, while the use of SABERTOOTH is advantageous for alignments at fold level. Our alignment scheme will profit from future improvements of structural profiles prediction.

**Conclusions:**

We present the automatic sequence alignment tool SABERTOOTH that computes pairwise sequence alignments of very high quality. SABERTOOTH is especially advantageous when applied to alignments of remotely related proteins. The source code is available at http://www.fkp.tu-darmstadt.de/sabertooth_project/, free for academic users upon request.

## Background

Protein alignment tools are key in many protein science applications. For very closely related proteins the alignment problem can easily be solved as a fuzzy string search in the sequences. However, with growing evolutionary distance more sophisticated techniques have to be applied to detect similarities. At about 25% sequence identity, when the alignment problem enters the so-called 'twilight zone' [1], related and unrelated proteins cannot be distinguished reliably by sequence based measures. A commonly applied strategy for this regime consists in exploiting not only the sequences that are to be aligned, but also evolutionary background information extracted from large sequence databases. This is achieved through analysis of multiple alignments from which one can obtain a suitable statistical description of the corresponding protein family. Especially for very distantly related sequences, the site-specific amino acid profiles or position-specific scoring matrices (PSSMs) obtained in this way increase the reliability of alignments by taking into account their evolutionary context.

Since protein structure evolves much more slowly than sequence, protein structure alignments are usually much more accurate for distantly related proteins and, hence, preferable if structural data is available. However, for the majority of cases only sequence information is available. A possible strategy for such cases would be to predict the unknown structures from their sequences and to perform a structure alignment using these predictions. Unfortunately, the accurate prediction of 3D coordinates is in general not possible yet and results are limited in quality [2]. Nevertheless, coarser descriptions of the protein structure than the one based on coordinates give sufficient information for many applications, and a number of algorithms exists that allow predicting structural characteristics of proteins such as secondary structure, residue-wise contact order, or solvent accessibility, to name but a few. Here we consider a reduced description of protein structures based on the contact vector (CV), whose *i*-th component represents the number of sites with which site *i *is in contact. The CV is strongly correlated with more complex descriptions such as solvent accessibility and effective connectivity of the contact matrix. We showed in previous work [3,4], that the CV, despite giving a very simplified description of a protein structure, is sufficient for obtaining state-of-the-art protein structure alignments.

## Results and Discussion

We previously developed the SABERTOOTH algorithm for performing protein structure alignments by aligning profiles that represent the protein structure [3,4]. In this work, we apply this algorithm to aligning structural profiles predicted from the protein sequence alone, thereby obtaining protein sequence alignments.

We adopted a structural profile based on the contact vector (CV) described above, which produces structural alignments of high quality and is simple to predict. Contact vector prediction is an active field of research: Bastolla *et al*. [5] described a very simple prediction method that defines typical hydrophobicity values per amino acid residue type assuming independent sites. The resulting hydrophobicities can be shown to correlate with typical CV components per residue type. A more elaborate approach is discussed by Vullo *et al*. [6] who employ an artificial neural network (ANN) to include correlations between amino acid sites along the sequence. An alternative approach to consider these correlations is described by Kinjo and Nishikawa [7] who run PSI-BLAST to compute position-specific scoring matrices (PSSMs) that are then input into a so-called critical random network (CRNPRED). The prediction scheme used here follows an idea introduced by Jones [8] to predict secondary structure propensities using an ANN with protein family information encoded in PSSMs. We modify this approach to predict the contact vector from protein sequence (see Methods).

The alignments produced by our algorithm following the strategy described above and detailed in the Methods section were then compared with some of the most commonly used sequence alignments programs, which we classified in three main classes: (1) Programs that take as only input the two sequences to be aligned; (2) Programs that take an additional input in form of a large database of known protein sequences and extract from it two families of proteins evolutionarily related with the query proteins; (3) Programs that, in addition to taking a sequence database as input, build a probabilistic description of the protein families as a Hidden Markov model (HMM). As reference tools of the first class we chose the programs Clustal W [9], MUSCLE [10], and T-Coffee [11]. These tools are commonly used to compute multiple sequence alignments. In the present work, we assess their performances in building pairwise alignments, since we compare them with our new pairwise algorithm. The construction of all-vs-all pairwise alignments is always the first step in building multiple alignments, and the accuracy of this first step strongly influences the final result. We expect that the quality achieved in the pairwise step will also benefit multiple alignments based on it. In addition, the construction of the multiple alignment depends on the choice of the set of sequences to be aligned. Influences of this set of sequences have to be ruled out in order to compare to the pairwise SABERTOOTH alignments. PSI-BLAST [12] was selected as a representative of the second class of programs, which make use of sequence databases to collect a large set of evolutionarily related proteins for each of the query sequences. SABERTOOTH and PSI-BLAST use the same input data, since SABERTOOTH uses PSSMs derived by PSI-BLAST as alignment parameters as well as for predicting the structural profiles, permitting a fair comparison between the two programs. As a representative of the third class of programs we considered the algorithm HHpred [13], which also uses as input the set of evolutionarily related proteins obtained through PSI-BLAST search and obtains from them a powerful probabilistic description of the protein family in terms of a Hidden Markov Model.

To compare the quality of sequence alignments, we adopted a new method that scores the structure similarity derived from alignments of proteins with known structure. In fact, measures of sequence similarity are not meaningful for distantly related proteins, and their use in the assessment could lead to circular results, since many sequence alignment algorithms are based on the optimization of sequence similarity. Moreover, for many practical applications, such as homology modelling and function prediction, sequence alignments are used to infer a structure alignment, which is the real aim. These arguments have motivated the practice to assess the quality of sequence alignments by using structure alignments as a 'gold standard', either computed by a structure alignment tool or taken from databases like BAliBase [14] or HOMSTRAD [15]. However, the problem to find the optimal structure alignment for a pair of proteins has no unique solution [16]. Existing algorithms differ in how they address this problem and have different biases that would inevitably influence the assessment based upon them. Therefore, the only way to obtain an unbiased assessment of alignment accuracy is to renounce using any supposed gold standard. However, this does not mean that we also have to renounce applying powerful structure similarity scores. Here we directly compute structure similarity scores from the sequence alignment. For instance, the optimal spatial superimposition corresponding to a given alignment can be obtained through the MaxSub algorithm [17], and the contact overlap can be directly computed from the alignment, without having to optimally superimpose the structures. In this way, we can objectively quantify the alignment quality without the need of a gold standard. As we show below, the qualitative results of a large scale comparison are quite clear, and they do not change when we use different structure similarity scores.

As an objective measure of structural similarity we adopt the contact overlap. We find this measure preferable to others because of three reasons: (1) It does not require to compute a structure superimposition, avoiding the influence of the choices necessary for defining an optimal rotation matrix; (2) It effectively weights more the sites that belong to the protein core due to the larger number of contacts found here; (3) In the important cases of conformation changes in which one subdomain of the protein moves with respect to the other one, for which superimposition-based measures underestimate the structural relatedness, the contact overlap only penalizes a small number of inter-subdomains contacts that do not match in the two structures, while superimposition-based scores also penalize intra-subdomain contacts. We also consider as additional similarity measure the TM-score [18], a well-known structural similarity measure based on structure superimposition. It is reassuring that the TM-score supports the results of the contact overlap. For comparative purposes, we also consider the sequence similarity measure. The definitions of these measures are given in the Methods section.

**Figure 1:**
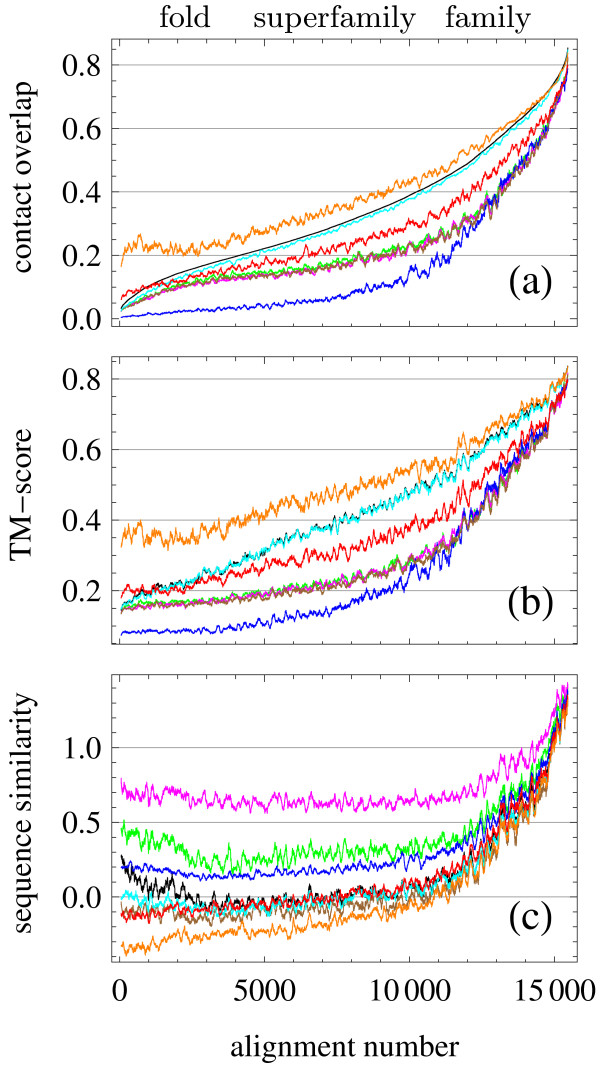
**Comparison of Alignment Accuracy in Terms of Contact Overlap, TM-score, and Sequence similarity**. Comparison of alignment accuracy measured in contact overlap, TM-score, and sequence similarity for SABERTOOTH (red), Clustal W (green), T-Coffee (brown), MUSCLE (magenta), PSI-BLAST on BLOSUM62 (blue), and HHpred (cyan). The black curves mark the 'best of' set. All alignments are sorted by increasing contact overlap of the 'best of' set from left to right. The abscissa is the resulting alignment number and the ordinates indicate the respective similarity measure with a running average of window size 100 applied to improve readability. SABERTOOTH structure alignment (orange) is added for illustrative purposes, but it is not included in the comparison. The SCOP level labels shown do not represent sharp borders, since the real assignments can overlap in this depiction.

In Fig. 1, we report the structural quality of over 15 thousand alignments assessed through the similarity measures described above. As a reference set we use a representative set of pairs of related proteins from the ASTRAL40 database of structural domains [19]. These are challenging alignments below the threshold of 40% sequence identity, ranging from the family level of strong evolutionary and probable functional relationships, through the superfamily level of more elusive evolutionary relationships, to the fold level of proteins structurally but not necessarily evolutionarily related. Curves in the plot depict the similarity measures of the alignments obtained through SABERTOOTH (red) and through the reference tools. For each pair of proteins, we select the alignment with largest contact overlap produced by all reference tools ('best of' set) and use the corresponding contact overlap to rank the alignments from difficult to easy ones. Therefore, the left part of the plots corresponds to distantly related pairs of proteins and the right part corresponds to closely related pairs. Loosely speaking, we call the left, middle and right part of the plots the "fold", "superfamily" and "family" range of similarity, respectively. Nevertheless, not all of the alignments included in each range belong to the corresponding SCOP level. For purposes of comparison, we also represent the best similarity measure produced by all reference tools ('best of' set) and the one produced through SABERTOOTH structure alignment by making direct use of structural information. The ranking of the alignment algorithms is consistent over the three ranges of similarity, with important differences discussed below, and using both structure similarity measures, as one can see comparing the top and middle plot of Fig. 1. In particular, PSI-BLAST produces the least accurate alignments except in the family region, where it is comparable or slightly better than the three references Clustal W, T-Coffee, and MUSCLE. The decrease of contact overlap and TM-score for PSI-BLAST is partly an artifact of the reduced percentage of aligned residues that result from the fact that PSI-BLAST outputs only the aligned segments that it considers relevant. The use of different substitution matrices with PSI-BLAST does not change this picture, in fact, we found only very slightly different results for BLOSUM45, BLOSUM62, and BLOSUM80, so that in the plots we only show results based on BLOSUM62. The three references Clustal W, T-Coffee, and MUSCLE are very similar in quality over the whole range of structural similarity, and their placing in the ranking is just above PSI-BLAST. Then we find SABERTOOTH, whose sequence similarity scores are better than those of the above mentioned tools except for very high similarity, where basically all tools achieve a similar quality. HHpred is unambiguously identified as the tool producing the best quality alignments over the whole relatedness range, and it even approaches the accuracy of structure alignment algorithms. One sees that the best reference almost exactly coincides with the HHpred results. Nevertheless, for very distantly related alignments SABERTOOTH reaches a quality even higher than that of HHpred. The mean values of the structural quality measures for alignments in the family, superfamily and fold range are reported in Table 1 for all programs. The lists of the alignments used for quality assessments at SCOP family, superfamily and fold level are reported in the Additional file 1.

**Table 1:** Comparison of Alignment Accuracies in Contact Overlap, TM-score, and Sequence Similarity

Program	family	superfamily	fold
SABERTOOTH (struct)	53.7/66.9/35.7	30.2/44.5/-13.9	28.4/42.4/-30.6
'best of'	50.1/62.3/40.0	26.8/36.3/-1.2	21.4/29.4/-6.8
HHpred	49.5/62.1/37.5	26.2/36.6/-4.3	20.1/29.2/-10.9
SABERTOOTH (seq)	43.8/55.6/45.7	20.6/29.7/-0.8	18.0/26.9/-12.1
Clustal W	37.0/47.2/55.9	17.0/21.6/22.0	14.8/19.8/18.4
PSI-BLAST	36.5/48.1/51.5	8.50/16.3/15.2	3.7/9.7/13.1
MUSCLE	36.3/47.4/86.1	16.1/21.7/63.5	13.4/19.1/56.7
T-Coffee	35.8/46.6/36.7	16.5/21.2/-7.4	13.8/18.4/-14.8

The table shows the length weighted mean values of contact overlap, TM-score, and sequence similarity (all values multiplied by 100) corresponding to Fig. 1 separated for the three test sets on the major SCOP similarity levels family, superfamily, and fold. The table is sorted by contact overlap on family level. SABERTOOTH structure alignment is added for illustrative purposes, but it is not included in the comparison.

In the bottom plot in Fig. 1 we show results of sequence similarity, maintaining the same colouring scheme of the tools and the same ordering of the alignments as in the plots above. The sequence similarity values agree with the contact overlap and the TM-score, in the sense that, for each tool, alignments endowed with larger structure similarity also display higher sequence similarity, as expected. However, the ranking of the tools is now completely different. The structure alignment provides the lowest sequence similarity, and the two algorithms that yield the highest structure similarity, namely HHpred and SABERTOOTH, output the lowest sequence similarity measures, together with T-Coffee. The three tools that show nearly identical performances in matters of contact overlap display very different sequence similarities. MUSCLE finds unrealistically high sequence similarity values still for very distantly related pairs deep in the fold level region. Also Clustal W and PSI-BLAST assign positive values in this region, in contradiction to SCOP's definition of the fold level. Surprisingly, HHpred's sequence similarity values slightly increase again for very low similarities which might reveal a tendency to over-optimisation of spurious sequence similarities in this area.

Comparing the results in matters of sequence similarity to those found with SABERTOOTH structure alignment [3,4] we can see that SABERTOOTH, HHpred and T-Coffee assign more realistic sequence similarity values than the other tools that cross zero sequence similarity in the early superfamily area. This analysis points out that all tools tend to overestimate sequence similarity with respect to structure alignment, whose quality is unambiguously better as measured in terms of contact overlap or TM-score and systematically lower when measured in terms of sequence similarity. This behaviour reveals an inherent bias of sequence alignment tools in this respect. This tendency is even more pronounced considering that the structure alignment of SABERTOOTH yields higher sequence similarities than other well known alignment tools [20].

As a second test, we evaluate the ability of the alignment programs to detect relevant relationships between the aligned proteins based on the significance measures they output. This is an important application, since sequence alignments are often used to predict whether two proteins share a similar function, have a close evolutionary relationship, or a similar structure, so that e.g. one can be used as a template for the other. As a test set of relevant relationships, we again consider the SCOP classification at family, superfamily and fold level. Although the SCOP classification is not fully consistent with quantitative measures of structural similarity, it provides a large number of mostly valid relationships that constitute a suitable benchmark.

**Figure 2:**
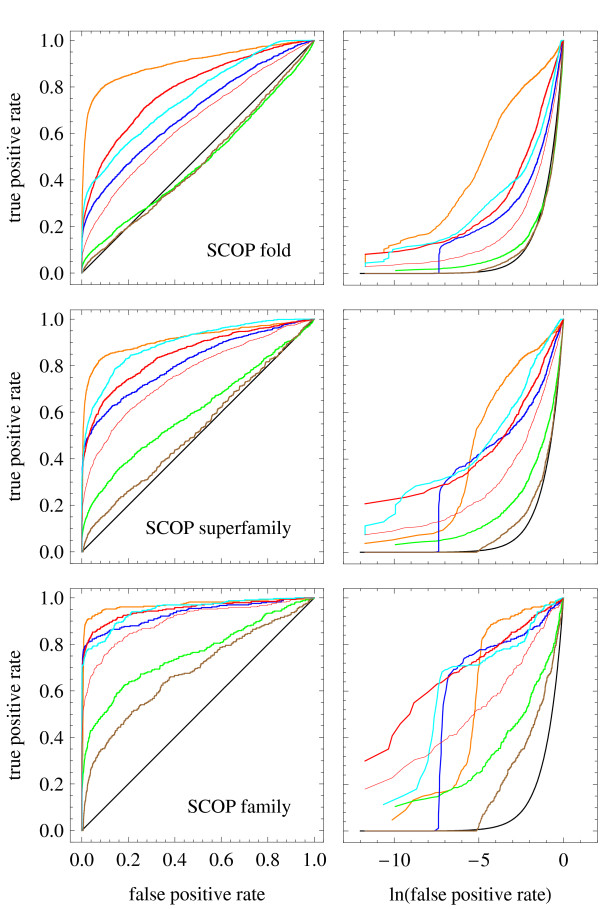
**Comparison of Significance Scores' Compliance with SCOP**. ROC plots for prediction of SCOP fold, superfamily and family relationships using the significance scores output by different programs. In the left panels the ROC plots are shown in standard linear scale, in the right panels the ROC plot are shown in log-linear scale in order to better appreciate the behaviour at low false positive rate. The colour code is as follows: SABERTOOTH (red), the thick line refers to the r_PSSM,B62 _based *Z*-score, while the thin line is relative to the seqSim_B62 _based *Z*-score, Clustal W (green), T-Coffee (brown), PSI-BLAST on BLOSUM62 (blue), and HHpred (cyan). SABERTOOTH structure alignment (orange) is added for illustrative purposes, but it is not included in the comparison.

The results of this analysis mostly agree with the first test, as shown in the ROC plots in Fig. 2. The larger the area under the curve of the ROC plot, the better the ability of the corresponding significance score to identify relationships at the given level. The structure alignments have the best performance at all levels, with their advantage with respect to sequence alignment growing from the family level to the fold level. However, not even structure similarity is perfect at detecting SCOP relationships, which is due to the fact that SCOP is an expert classification not entirely consistent with structure similarity measures. Of the sequence aligners, the best performances are once again obtained by HHpred at superfamily level, with SABERTOOTH's significance score consistently performing second-best and PSI-BLAST being the third best, despite its structure similarity measure being lower than that of Clustal W, MUSCLE and T-Coffee. However, SABERTOOTH gets almost equal to HHpred at family level, and performs slightly better than it at detecting relationships at fold level, consistent with SABERTOOTH's improved structure similarity in this range. This is consistent with the insight that alignment quality as assessed from structure and on evolutionary relationships are intimately related. The output sequence similarity, in contrast to that, is unrelated with the ability to detect significant evolutionary relationships. We also show in Fig. 2, right panels, the ROC plots in log-linear scale in order to focus on the region of very low false positive rate. It can be seen that, surprisingly, for the family and the superfamily level the significance scores derived from structure alignments have a lower true positive rate at very low false positive rate than those derived from sequence alignments. Numerical values for the area under curve in the ROC plots can be found in Table 2. The list of the 123,753 alignments used for significance score assessment is reported in Additional file 2.

**Table 2:** Comparison of the Compliance of Significance Scores with the SCOP Classification

Program	family	superfamily	fold
SABERTOOTH (struct)	96.85	91.54	90.29
SABERTOOTH (seq)	95.05	85.06	79.04
HHpred	94.84	89.55	75.69
PSI-BLAST B62	93.22	81.32	68.99
PSI-BLAST B80	92.53	81.88	69.74
PSI-BLAST B45	91.64	80.70	69.21
SABERTOOTH (seq/seqSim)	89.96	76.16	64.61
Clustal W	75.79	60.52	47.96
T-Coffee	68.01	52.87	47.65

The table shows the area under curve (AUC) in percentage for all programs and SCOP levels shown in Fig. 2, sorted by the first column. For 'SABERTOOTH (seq/seqSim)' significance is measured using the less accurate seqSim instead of the PSSM score, data is added for illustration. SABERTOOTH structure alignment is added for illustrative purposes, but it is not included in the comparison.

**Figure 3:**
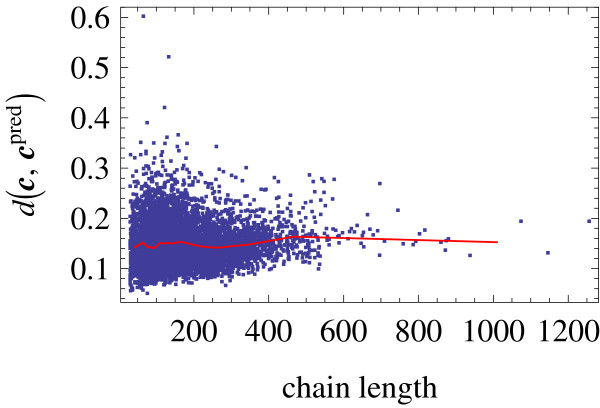
**Contact Vector Prediction Quality**. The contact vector prediction quality is shown, plotting the penalty for aligning the corresponding observed contact vectors, , which is relevant for the alignment algorithm, versus chain length. The figure shows a dot for each domain in the ASTRAL40 set of 9420 protein domains. The correlation of this quantity with chain length *r *= 0.0516 is rather weak.

Finally, we assessed the accuracy of the prediction of the structural profile that SABERTOOTH uses for sequence alignment. The predicted profile was compared with the profile measured over a test set of 9420 protein domains, disjoint from the training set used. For each domain, we measured the correlation between the predicted and the measured contact vector, finding a length weighted correlation coefficient of *r*(CV, predCV) = 0.72. This comparison is in part affected by the fact that single chains and single domains are considered in the test set, so that the observed number of contacts does not include inter-chain and inter-domain contacts, thus underestimating the observed number of contacts with respect to the case in which the whole multi-chain protein is considered. We also measured the variance of the predicted contact vector for each domain, and compared it with the observed variance. The predicted variance is systematically underestimated and not very well predicted, as indicated by the weak correlation coefficient *r*(var_CV_, var_predCV_) = 0.385. This suggests that it is still possible to improve the performance of SABERTOOTH by improving the prediction of the contact vector. The quantity relevant for the alignment algorithm is the penalty that has to be paid for aligning the observed and predicted contact vector,  (see Methods). We measured this quantity for each domain in the test set, and plotted it versus chain length in Fig. 3. Each point in the plot represents a protein domain from the test set. One can see that the penalties are low and that they are almost uncorrelated with chain length.

## Conclusions

The SABERTOOTH algorithm can align vectorial representations of protein structures. This was first applied to cases in which the protein structures are known, so that the algorithm produces a structure alignment. In the present paper, we apply the same algorithm to aligning vectorial representations predicted based on sequence alone, obtaining high quality sequence alignments. This new approach to sequence alignment is made possible because we adopt a very simple structural representation, the contact vector, which can be predicted relatively easily based on sequence, and it presents three main advantages: First, the predicted contact vectors are expected to capture structural features that are more conserved in evolution than the sequence itself, which leads to an increased sensitivity when comparing remotely related proteins. Second, we perform sequence alignment by aligning the same structural profiles that we adopt for structure alignment, which enables us to treat structure and sequence alignment with the same formalism and the same algorithm. Third, sequence alignment quality is expected to further improve through improvements in the profile prediction scheme and adding other types of structure description such as secondary structure. As a caveat, we warn that the application of SABERTOOTH should be limited to proteins predicted to be globular, since the strategy that we apply is not suitable for the alignment of disordered proteins.

Our results show that the approach to combine structural profile prediction from sequence with our generic alignment program SABERTOOTH leads to sequence alignment quality better than those of most widely used algorithms we compare with, with the exception of HHpred, which shows the best performance but is also computationally more expensive than SABERTOOTH. It is interesting to notice that SABERTOOTH shows slightly better quality than HHpred for aligning very distantly related pairs, a difficult and important task for the field of homology modelling. Therefore, the use of SABERTOOTH is anticipated to be advantageous at least for these difficult alignments, whereas for less distant alignments users should balance the better quality of HHpred with its heavier computational burden. These improved performances for distantly related alignments can exploit the fact that protein structure is more conserved in evolution than protein sequence, and that the alignment algorithm and parameters used were designed and trained to align contact vectors derived from coordinates. This reduces the bias to overemphasize sequence similarity, which tends to become insignificant at large evolutionary distance, and which is overestimated by most other sequence alignment algorithms. In fact, comparing the quality of the structural similarity measure and the sequence similarity measure for various sequence aligners, we see that the alignment algorithms that output the highest sequence similarity in the region of distantly related proteins also tend to produce the worst results in terms of structure similarity. This tendency is particularly clear when structural aligners are included in the comparison. Moreover, three reference methods output alignments that are of very similar quality as assessed through their structural similarity score, but their sequence similarity scores yield very different values. This implies that absolute sequence similarity values depend strongly on the algorithm used and are therefore not very informative for quantifying the relatedness of distantly related proteins. Consistently, the performances of these algorithms for recognising distant evolutionary or structural relationships are comparable or even worse than random, as the ROC analysis shows. This suggests that sequence similarity values output by sequence alignment programs are not very meaningful for distantly related proteins, and they are not comparable between different programs.

The improved alignment accuracy has a cost in computational complexity. The runtime of our alignment routine cannot compete with highly optimized tools even when we pre-compute the profile predictions. While the runtime of the neural network for the prediction of the profiles can be neglected, the computation of the PSSM by PSI-BLAST is quite lengthy, heavily depending on the number of amino acids in the query sequence, PSI-BLAST parameters, and the sequence database used. The runtime of the alignment routine with pre-computed profiles takes approximately as long as the fastest structure alignment tools existing today. Including the computation of the profiles, SABERTOOTH and PSI-BLAST are comparable in computation time, while HHpred is about three times slower due to the larger number of iterations used to compute the PSI-BLAST PSSMs that also underly HHpred's alignments. Taking accuracy and computation time into account, we envisage the application of SABERTOOTH to studies in which a large number of sequence alignments have to be performed, so that HHpred and similar methods would be too costly, but high accuracy is nevertheless required, and to studies that deal with the comparison of very distantly related proteins. Furthermore, there is still room for improving SABERTOOTH in two directions. First, it is possible to improve the quality of the contact vector predictions. If these were perfect, the quality of the produced alignments would be comparable to those obtained with HHpred. Second, it is possible to consider structural information such as predicted secondary structure. Finally, SABERTOOTH facilitates analyses that make use of different types of alignments, since the same program can be used not only for sequence alignment but also for structure alignment [4] and sequence-to-structure alignment without changes. The latter application will be treated in a later publication.

## Methods

### Structural Representation

In this work, we use the contact vector  as structural representation of the protein, where *N *is the length of the protein sequence, and the contact matrix *C*_*ij *_is a binary symmetric matrix with components equal to one if the corresponding amino acids are in contact in the 3D structure and zero otherwise. Therefore, the contact vector counts the number of contacts that site *i *has with all other sites in the protein. For convenience, the structural profile that we align consist of the normalized contact vector *c*_*i*_, defined as(1)

where ⟨*v*_*i*_⟩ = ∑_*i*_*v*_*i*_/*N*, so that its average value is one for all proteins.

Here we define a contact if the C_*α *_atoms of residues *i *and *j *are closer than *d*_th _= 17 Å Pairs of sites *ij *with |*i *- *j*| < 3 correspond to non-informative contacts, and their *C*_*ij *_are explicitly set to zero. These parameters were selected because their performances are close to optimal for structural alignments [4]. The normalized contact vector's component *c*_*i *_represents the contact density or connectivity of amino acid *i *within the structure. For our purpose here, we aim to predict the contact vector using an artificial neural network approach. The prediction scheme is discussed in more detail below.

### SABERTOOTH Alignment Algorithm

The algorithm minimizes a penalty function based on commonly used substitution penalties as well as insertion and extension of gaps. The alignment of two amino acids is penalized by the difference of the sites' structural descriptors, plus a contribution for amino acid substitution. Opening gaps is penalized by a term that depends on the components of the structural vector between which the chain has to be 'broken', while extending a gap is modelled by summing over all components opposite that gap. The algorithm, the penalty function, and parameters to perform sequence alignments are identical to those used already for structure alignments before [3,4].

All possible alignments output by SABERTOOTH can be created by inserting an arbitrary number of gaps of arbitrary lengths into one or the other sequence in the alignment. In this way the sequences stay intact and repetition, mirroring, or exchange of fragments are not permitted. All alignments complying with this definition can be displayed as paths through an alignment matrix *A*_*ij*_, analogous in shape to a dot matrix, that start in the top row (or left column) and end in the right column (or bottom row). In the steps though *A*_*ij*_, moving to the right (down) inserts or extends a gap in sequence 1 (sequence 2) while moving right-down in a diagonal step depicts alignment of the respective amino acids. A penalty is added if amino acids of different connectivities are aligned, i.e. component values of the contact vector, and/or different amino acid residue types, and for the insertion or extension of gaps. The penalty function is divided into four terms that depend on the contact vectors *c*_*i *_and *c*_*j*_, and the protein sequences *A*_*i*_, with *A*_*i *_one of the twenty natural amino acids types.

Aligned components of the structural profiles, corresponding to position *i *(*j*) in the first (second) profile, are penalized by a term *M*_*ij *_that grows with their absolute difference raised to a suitable power  whose value was optimized in a previous study,(2)

Substitution of amino acids is less likely for pairs with very different physiochemical properties just like used in sequence alignments,(3)

with the parameters  and  and with  for the substitution probability connecting amino acids  and . The substitution probabilities were recovered from BLOSUM62 using the program *lambda *by Eddy [21].

Breaking chain *s *between residues *i *and *i *+ 1 is penalized by(4)

with parameters  and  and with *s *∈ {1,2} labelling the chain into which the gap is inserted.

An insertion of length *n*_*j *_into chain *s *at position *j *+ 1 opposite to a gap in the other chain, consisting of the components , is penalized by(5)

with parameters  and  and with *s *∈ {1,2} selecting the chain.

The total penalty function *F *combines all these contributions,(6)

where  is the set of all aligned pairs of amino acids, ℬ^(*s*) ^the set of all positions *i *after which chain *s *is broken, and ℐ^(*s*) ^the set of all insertions of length *n *after position *j *in chain *s*. The alignment result is determined by the path through alignment matrix *A*_*ij *_that globally minimizes penalty function *F*, Eq. (6). It corresponds to the optimal alignment, given penalty parameters and proper definition of *F*. The parameters used in *F *are shown in Table 3. They were optimized for structure alignment and have not been changed for the present purpose.

**Table 3:** Parameters used in Alignment Penalty Function

Name	Factor	Exponent
*p*_align_	1.	1.20648
*p*AA_*subst*_	0.5947	11.11090
*p*_break_	1.0964	1.60294
*p*_insert_	0.5025	2.28409
*p*_insert@term_	0.3360	2.28409

The table shows the alignment penalties used for structure and sequence alignments by SABERTOOTH. The values are relative to alignments using the contact vector defined on C_*α *_distances with contact threshold *d*_th _= 17 Å and three suppressed diagonals *n*_D _= 3. These are the same parameters used in [4] for structure alignment.

Different algorithms can be used to find this optimal path very efficiently, our implementation uses Dijkstra's shortest path algorithm. A more detailed description of the full alignment algorithm is given in [3], the specific implementation using the contact vector as structural profile is discussed in [4].

### Structural Profile Prediction

The CV is predicted here using an artificial neural network (ANN) scheme based on position-specific scoring matrices (PSSMs) computed by PSI-BLAST. Relying on PSSMs we follow an idea already employed by Jones [8] to predict secondary structure propensities.

Formally the PSSM components characterized probabilities propensities to find a specific amino acid of type *a *at site *i *of the given protein family, where the index *a *∈ {1, 20} labels the twenty natural amino acids. The matrix entries are defined as(7)

where *Q*_*i*_(*a*) quantifies the probability to find the amino acid *a *at site *i *in a sequence belonging to the same protein family and *P*(*a*) characterizes the background distribution, independent of the position in the sequence.

We used the non-redundant sequence database 'NR' from NCBI (6^th ^Feb. 2009) after filtering out biased regions using *pfilt *by Jones and Swindells [22]. The resulting database 'nrfilt' contains 5,884,546 sequences or 2,028,361,679 residues.

The ANN implements the very basic feed-forward scheme in which the flow of information is unidirectional, starting in the 15·21 neurons of the input layer that accounts for correlations along the sequence over a fifteen sites wide window, for each of the twenty amino acids and an additional terminal marker, leading through only one hidden layer with 40 neurons to the output layer consisting of a single neuron. An ANN with only one hidden layer can be described by a single non-linear function as(8)

with neuron state vector  and layer sizes *N*_1 _= 21·15 = 315 and *N*_2 _= 40.

The parameters *υ*_*ij *_and *ω*_*ij *_were trained on a set of about 3000 sequences randomly selected from rank-1 of a PDB clusters set at 50% sequence identity level. We excluded from this set all sequences that were not resolved by X-ray, that are transmembrane according to *pdbtm *[23] or shorter than 30 or longer than 300 residues. Furthermore, non-globular structures were sorted out by the *ad hoc *criterion introduced by Bastolla *et al*. [24]. We split the whole set into training and validation sets of equal sizes. For parameter training we used online learning, minimising the quadratic distance between prediction and structure derived contact vector, in combination with early stopping. The ANN parameter window size in the input layer, number and size of hidden layer, choice of function and training criterion were selected by extensive testing. Cross-validation was used to make sure that the predicted parameters are not over-fitted.

The contact vector prediction scheme does not properly predict the scale of the output vectors, so that their mean values are not fixed and the variance of the vectors has only very low correlation with the targets. While the mean value can be set to one simply by dividing by the mean component value, the variance needs to be predicted independently. In fact, the variance is systematically underestimated by the predictor, a behaviour that can possibly be explained by the very broad distribution of contact values around their residue type specific means. The ANN tends to introduce a bias that shifts the predicted components to their mean values, attenuating the variance of the predicted vectors.

In order to compensate this effect we aim to predict the variance in an additional computation in dependence of amino acid composition and length of the sequence. The predicted variance value is than stamped on the formerly predicted vector by scaling its components. To do so, we rely on an ansatz similar to the one used by Kinjo *et al*. [25]. Sequence information enters the scheme through the 20 mean values, one for each amino acid residue type, computed over the given sequence-specific PSSM that was already used as input to the ANN. The length dependence of the variance is fitted by(9)

consisting of a term that describes the scattering of the variance (with the amino acid specific fit parameters *f*_*i *_and length parameter *a*) and by modelling the length dependent mean value of the variance as a power law (with parameters *b *and *c*). Furthermore, a lower bound value vãr_min _= 0.05 is used to suppress artificially low variance values.

### Assessment of the CV Prediction

We evaluated the accuracy of the predicted structural profiles by comparing the predicted CVs with those computed directly from protein structure. The test set comprises the whole ASTRAL40 (version 1.73) with 9420 protein domains.

### Assessment of the Alignment Quality

The quality of the alignments produced by SABERTOOTH and by the reference tools described below was assessed on three sets of alignments from the major levels of SCOP family (5238 alignments), superfamily (5180), and fold (5097). Pairs in the sets were chosen randomly from the ASTRAL40 subset of SCOP domains that have less than 40% pairwise sequence identity. The fold level test set consists of alignments from the same fold but different superfamilies, and the superfamily test consists of alignments from the same superfamily but different families. We assessed only the alignments for which all reference tools output results, which reduced the sets by about half a percentage in size. SABERTOOTH outputs alignments for all examples in all sets.

The alignment quality was quantified using as structural similarity measure the contact overlap *q *that quantifies the similarity between the contact matrices of two protein structures, normalized with the geometric mean of their number of contacts in such a way that *q *= 1 for identical contact matrices(10)

As an additional measure of structural similarity we used the TM-score [18] that quantifies the closeness of the protein's backbone atoms after optimal spatial superimposition  using a MaxSub rotation [17]. This measure is highly correlated to other similarity measures based on superimposition, such as the Percentage of Structural Identity (PSI) or the Global Distance Test (GDT) scores [26].

The sequence similarity(11)

with  the BLOSUM62 constitutes a local measure describing the precision in the details if contact overlap values of the same alignment are reasonably high.

### Definition of Significance Scores

SABERTOOTH outputs two different significance scores  implemented as *Z*-scores, assuming Gaussian distribution of the underlying scores ,(12)

The mean and the standard deviation of the scores that are used in the above formula depend on the lengths of the compared proteins *N*_1 _and *N*_2_. For simplicity, we consider in the following that this dependence only acts through the smaller of the two lengths, *N *= min(*N*_1_, *N*_2_). This length dependence is assumed to be of power-law type, and the parameters are obtained by fitting power-laws on a set of alignments of non-related proteins, consisting of 723,217 alignments of domains from different SCOP classes that were tested to have MAMMOTH [27]*Z*-score below 0.75 and TM-align TM-score [18] below 0.25 to make sure to exclude even distantly related proteins.

The first similarity score is derived from the same PSSMs already utilized for predicting the structural profiles. It is computed as a sum over Pearson correlation coefficients *r *of the PSSM columns for every pair of aligned sites, normalized by shorter chain length,(13)

with power-law fits resulting to(14)(15)

The second score is much less significant, and it is mainly kept for reasons of comparison. It is derived from simple sequence similarity, Eq. (11), computed using a BLOSUM62 and normalized on the length of the shorter chain. The power-law fits result to(16)(17)

### Assessment of the Significance Scores

With all tools, with the exception of MUSCLE that does not output any significance score, we computed significance scores for the all-vs-all alignments of 498 structures that were randomly selected from the 97 most populated folds in ASTRAL40 (version 1.73), leading to 123,753 alignments. Using these significance scores, we computed Receiver Operating Characteristic (ROC) curves that graphically quantify the agreement of the significance score with the SCOP classification. To create the ROC plots, a cutoff for the respective score *Z*_cutoff _is shifted from its minimum to its maximum value, counting the number of alignments with *Z *> *Z*_cutoff _at each value of *Z*_cutoff_. All alignments above the threshold that are also in the same cluster of SCOP, represent true positives TP, i.e. they agree with the classification. All those below the threshold but nevertheless in the same cluster of SCOP are counted as false positives FP that disagree with the classification. The assessment is split into independent analyses on the three major SCOP levels fold, superfamily, and family.

The ROC curves show the ratios of TP/P over FP/N, with the total numbers of positives P and negatives N, respectively. The further the curves reach to the left hand side of the plot, the less positives are assigned low values of significance, while pushing the curve up means that more positives are correctly assigned high significance. Hence, the larger the area under the curve (AUC), the better the performance of the significance score output by the respective program in recognising evolutionary and structural relationships given by SCOP. This can also be understood as measuring sensitivity and coverage. The diagonal marks the result for a random score.

### Sequence Alignment Reference Tools

We chose standard parameters for all alignment tools. Every modification in the parameters would infer previous knowledge about the set of sequences to be aligned. An expert for a specific program with in-depth information about the alignment set could, hence, achieve better results than found here. In this sense, we only assess the most general alignments but with fair chances for all algorithms. SABERTOOTH does not use any specific parameter set either, its sequence dependent alignment penalties rely on the all purpose substitution matrix BLOSUM62, and the profile prediction routine uses the identical PSI-BLAST PSSMs that were also used for the PSI-BLAST alignments.

#### Clustal W, T-Coffee, MUSCLE

All three multiple sequence alignment programs Clustal W (v1.83) [9], T-Coffee (v5.65) [11], and MUSCLE (v3.7) [10] start by computing the all-vs-all pairwise alignments of the input sequences. The resulting similarity scores are then used to build a tree that guides the order in which sequences are progressively aligned to build a multiple alignment. Here we only consider the initial pairwise alignments, whose quality strongly in uences the resulting multiple alignment.

Clustal W, T-Coffee, and MUSCLE were run with standard parameters. For Clustal W that implies in particular to compute 'SLOW' alignments with full dynamic programming for maximum accuracy. T-Coffee was used with the non-standard parameter '-do_normalize 0' to suppress normalisation of the output score which does not change the resulting alignment but improves the score's performance in the classification test.

#### PSI-BLAST

The alignment program PSI-BLAST (v2.2.18) [12] follows a fundamentally different strategy than the first three tools. It massively aligns the query sequence against a given database of sequences. After the first round of one-vs-all alignments the used substitution matrix is modified in order to better fit the query sequence in the next iteration. This recipe can be repeated several times refining the so-called position-specific scoring matrix (PSSM). The crucial points are the similarity measure and cutoff value, as well as the number of iterations that define the set of sequences that contribute to the PSSM. PSI-BLAST is designed to achieve much improved alignment quality especially for distantly related sequences in comparison to the other references.

Since PSI-BLAST aligns a single sequence to a database its alignments are intrinsically asymmetric, depending on the choice of query sequence. To eliminate this ambiguity in our application we perform two separate alignments. After computing PSSMs for both sequences, we align sequence *A *with a database containing sequence *B *using the PSSM computed for *A*, and vice versa. The alignment with higher PSI-BLAST E-value is selected as the final alignment referred to in both evaluations. We found that this choice leads to improved performance in the detection of evolutionary relationships than selecting the alignment with lower E-value or just a random one, as expected. Note that this procedure gives PSI-BLAST a slight advantage over all other tools evaluated here.

In order to assess the influence of the substitution matrix employed, we used PSI-BLAST with three different matrices: (1) BLOSUM62 with gapopen = 11 and gapextend = 1, the standard parameter set, (2) BLOSUM45 with gapopen = 14 and gapextend = 2, and (3) BLOSUM80 with gapopen = 10 and gapextend = 1. The gap penalty values relative to the choice of BLOSUM were taken from the PSI-BLAST help on EBI's web page at http://www.ebi.ac.uk/Tools/PSIBLAST/. It turned out that results found with the different BLOSUMs differ only very slightly. The results shown are for BLOSUM62.

#### HHpred

HHpred (v.1.5.0) constructs Hidden Markov Models (HMM) from alignments of the non-redundant database 'NR' from NCBI against a query sequence. These alignments are obtained by PSI-BLAST. HHpred then computes the final alignment, maximising a score based on the coemission probability of the two HMMs and predicted secondary structure. For the alignment overlap test, we used the global alignment mode of HHpred while we used the local alignment mode for the fold recognition test, thus yielding the best results for the respective analysis. The local alignment mode performs worse in the alignment overlap test because it aligns residues in the conserved core, only. Please note that this proceeding is to the advantage of HHpred in comparison to all other alignment tools used here, since all other tools use identical sets of alignments for both tests.

### Data Sets

#### Alignment Quality Assessment

The data set used for alignment quality assessment consists of a random selection of pairs from the ASTRAL40 subset of SCOP domains that have less than 40% pairwise sequence identity. It contains three sets of alignments from the major levels of SCOP family (5238 alignments), superfamily (5180), and fold (5097). All alignments from the different similarity levels are from different clusters of the underlying level, i.e. alignments from the same fold are from different superfamilies and so on.

#### SABERTOOTH Significance Score Training

Statistics over 723,217 alignments, randomly selected from different SCOP classes, were used to fit the length dependent mean and standard deviation values used in the SABERTOOTH significant scores. Alignments with MAMMOTH [27]*Z*-score below 0.75 or TM-align TM-score [18] below 0.25 were sorted out to make sure that all examples represent alignments of reasonably unrelated proteins.

#### Significance Score Assessment

The 123,753 alignments underlying the ROC curve analyses include the all-vs-all alignments of 498 structures that were randomly selected from the 97 most populated folds in ASTRAL40 (version 1.73).

#### Contact Vector Prediction

For the contact vector prediction we selected at random about 3000 sequences from a PDB clusters set at 50% sequence identity level (rank 1) with chain lengths between 30 and 300 residues. Only X-ray resolved structures not classified as transmembrane according to *pdbtm *[23] were accepted. Furthermore, non-globular structures were sorted out by the *ad hoc *criterion introduced by Bastolla *et al*. [24].

#### Contact Vector Prediction Assessment

For the assessment of the contact vector prediction the whole ASTRAL40 (version 1.73) database with 9420 protein domains was applied. This test set is disjunct from the training set used.

## Availability and Requirements

Project name: SABERTOOTH Sequence Alignment

Project home page: http://www2.fkp.tu-darmstadt.de/bioinf/sabertooth_project/

Licence: Source code available on request to academic users, free of charge.

## Abbreviations

ANN: Artificial Neural Network; AUC: Area Under Curve; BLOSUM: BLOcks SUbstitution Matrix; CRN: Critical Random Network; CV: Contact Vector; HMM: Hidden Markov Models; PSI: Percentage of Structural Identity; PSSM: Position-Specific Scoring Matrix; ROC: Receiver Operating Characteristic; SCOP: Structural Classification Of Proteins.

## Authors' contributions

MP and UB designed research; FT and MP developed the alignment algorithm; FT coded and tested the alignment algorithm; MP and JM developed the profile prediction scheme; JM coded and tested the profile prediction scheme; FT, JM, UB, and MP analysed results; FT, JM, UB, and MP wrote the paper. All authors read and approved the final manuscript.

## Supplementary Material

Additional file 1 **Alignment Quality Test Set**. The zip archive includes the three test sets used for alignment quality assessment in this work: 5238 alignments at SCOP family level, 5180 alignments at SCOP superfamily level and 5097 alignments at SCOP fold level. Each line identifies an alignment through two domain identifiers relative to SCOP version 1.73. PDB-formatted files for these domains can be found in the ASTRAL40 domain database version 1.73 at http://astral.berkeley.edu/. A more detailed description of the composition of the test sets is given in the Methods section. Click here for file

Additional file 2 **Significance Score Assessment Set**. The file contains the set of 123,753 alignments used for significance score assessment in this work. The alignments comprise the all-vs-all combination of 497 ASTRAL40 domains (version 1.73). Each row describing one alignment, the columns list the following information that allows retracing the SCOP classification: ASTRAL identifiers for first and second domain in the alignment, chain length of the first and the second. After that, in pairs of columns relative to the first and the second domain, the SCOP classification is listed: class (cl), fold (cf), superfamily (sf), family (fa), domain (dm), and species (sp) identifiers. A more detailed description of the composition of the set is given in the Methods section. Click here for file
